# Hemoptysis in Renal Transplant Recipients

**DOI:** 10.15388/Amed.2021.28.2.4

**Published:** 2021-07-29

**Authors:** Vasiliki Epameinondas Georgakopoulou, Aikaterini Gkoufa, Aikaterini Aravantinou, Nikolaos Garmpis, Konstantinos Mantzouranis, Serafeim Chlapoutakis, Pagona Sklapani, Anna Garmpi, Nikolaos Trakas, Christos Damaskos

**Affiliations:** Pulmonology Department, Laiko General Hospital, Athens, Greece; First Department of Internal Medicine, Laiko General Hospital, Medical School, National and Kapodistrian University of Athens, Athens, Greece; First Department of Internal Medicine, Laiko General Hospital, Medical School, National and Kapodistrian University of Athens, Athens, Greece; Second Department of Propedeutic Surgery, Laiko General Hospital, Medical School, National and Kapodistrian University of Athens, Athens, Greece N.S. Christeas Laboratory of Experimental Surgery and Surgical Research, Medical School, National and Kapodistrian University of Athens, Athens, Greece; Second Department of Propedeutic Surgery, Laiko General Hospital, Medical School, National and Kapodistrian University of Athens, Athens, Greece; Department of Thoracic Surgery, Agios Savvas Hospital, Athens, Greece; Department of Cytology, Mitera Hospital, Athens, Greece; First Department of Propedeutic Internal Medicine, Laiko General Hospital, Medical School, National and Kapodistrian University of Athens, Athens, Greece; Department of Biochemistry, Sismanogleio Hospital, Athens, Greece; N.S. Christeas Laboratory of Experimental Surgery and Surgical Research, Medical School, National and Kapodistrian University of Athens, Athens, Greece Renal Transplantation Unit, Laiko General Hospital, Athens, Greece

**Keywords:** Hemoptysis, Pulmonary Hemorrhage, Renal Transplantation

## Abstract

**Background:**

Hemoptysis is the expectoration of blood from respiratory system. Clinical entities associated with hemoptysis in renal transplant recipients differ from those causing hemoptysis in common population. This review summarizes all reported clinical conditions related to hemoptysis in renal transplant patients, their causes, diagnostic approach and management.

**Methods and Materials:**

Primary literature was researched through MEDLINE/PubMed database and Google Scholar without restrictions. Relevant and current literatures related to hemoptysis in renal transplant recipients were used.

**Results:**

Overall 23 articles were included, since they report clinical conditions associated with hemoptysis in renal transplant recipients. Opportunistic lung infections, pulmonary hemorrhage associated with drugs and malignancies are usual causes of hemoptysis in these patients, sometimes with a fatal outcome.

**Conclusions:**

Hemoptysis is an important manifestation in renal transplant patients, caused frequently by rare clinical entities, different from those in common population.

## Abstract (lt)

**Inkstų transplantatų recipientų hemoptizė:**

**Santrauka. *****Problema. ***Hemoptizė – atsikosėjimas krauju. Klinikiniai hemoptizės atvejai, nustatyti inkstų transplantatų recipientams, skiriasi nuo hemoptizės atvejų, nustatomų visoje populiacijoje. Šioje apžvalgoje pateikiamos visos klinikinės būklės, susijusios su pacientų, kuriems persodinti inkstai, hemoptize, jų priežastys, diagnostikos būdas ir gydymas.

***Metodai ir medžiaga.*** Pirminės literatūros šaltinių ieškota MEDLINE / „PubMed“ duomenų bazėse ir „Google Scholar“ be apribojimų. Buvo naudojami aktualūs ir šiuolaikiniai literatūros šaltiniai, susiję su inkstų transplantatų recipientų hemoptize.

***Rezultatai.*** Iš viso į tyrimą įtraukti 23 straipsniai, kuriuose aprašomos klinikinės būklės, siejamos su inkstų transplantatų recipientais. Dažniausiai hemoptizė šiems pacientams pasireiškia ir kartais yra mirtina dėl atsitiktinių plaučių infekcijų, plaučių hemoragijos, siejamos su vaistų vartojimu ir vėžiniais susirgimais.

***Išvados.*** Hemoptizė pasireiškia inkstų transplantatų recipientams, ją dažnai sukelia reti klinikiniai atvejai, kurie skiriasi nuo atvejų, būdingų visai populiacijai.

**Raktažodžiai: **hemoptizė, plaučių hemoragija, inkstų transplantacija

## Introduction

Hemoptysis is the expectorating of blοod or blood-stained mucus from the respiratory system. Common causes of hemoptysis in developed countries include lung cancer, bronchiectasis and acute bronchitis, while in developing countries *Mycobacterium tuberculosis* and *Paragonimus westermani* lung infections and noncystic fibrosis (CF) related bronchiectasis are more frequent causes [1]. In addition, bleeding disorders such as thrombocytopenia, disseminated intravascular anticoagulation, platelet dysfunction syndromes and von Willebrand disease) and use of anticoagulant or antiplatelet medications may lead to non-life-threatening or life-threatening hemoptysis [2-4].

The lung parenchyma is mostly supplied by the low-pressure pulmonary artery circulation, from which most episodes of hemoptysis are originated. In contrast, the bronchial artery circulation is a high-pressure circulation, originating from the systemic circulation, that supplies mostly the endobronchial tree. Hemorrhage from a bronchial artery is the cause of life-threatening hemoptysis in the most of the cases, although the bronchial artery circulation is responsible for only 2% of the total lung supply [5].

Renal transplant recipients, as immunocompromised hosts, present pulmonary complications, mostly infections, like invasive aspergillosis, with hemoptysis as a characteristic manifestation, sometimes with a fatal outcome. In addition, medications and localized causes of lung hemorrhage like malignancies must also be considered in differential diagnosis of hemoptysis in these patients [6]. This review summarizes all reported clinical conditions associated with hemoptysis in renal transplant patients. It also summarizes their causes, that differ frequently from common causes of hemoptysis and their diagnostic approach.

## Methods and Materials

An electronic search in MEDLINE/PubMed and Google Scholar was conducted with the keywords “Hemoptysis” AND “Renal Transplant Recipients” OR “Kidney Transplant Recipients” OR “Renal Transplant Patients” OR “Kidney Transplant Patients” with English language limitation. All articles were accurately screened by two authors, and those reporting data on hemoptysis in renal transplant recipients were included. The reference list of each document that could be identified with our criteria was also hand-searched for detecting other eligible studies. 

## Results 

Overall, 100 articles were originally identified using our search criteria and from the reference list of the previously identified documents. Following elimination of duplicates, 77 were excluded after screening of title, abstract or full text, since they did not report the occurrence of hemoptysis in renal transplant recipients or they were not written in English language. Table 1 summarizes the studies describing clinical conditions associated with hemoptysis in renal transplant recipients, their causes, diagnostic approach, and management. Lung infections, pulmonary hemorrhage associated with drugs and malignancies are usual causes of hemoptysis in these patients. Imaging with chest X-ray and computed tomography (CT), bronchoscopy and microbiological evaluation of body fluids are important diagnostic tools.

**Table 1.** Studies describing clinical conditions associated with hemoptysis in renal transplant recipients, their causes, diagnostic method and therapeutic approach.

Study	Age/Sex	Causative Agent	Diagnostic Method	Outcome
Lattes et al. [6]	N/A	Pulmonary Tuberculosis	Sputum Culture	Death
Ardalan et al. [7]	32/M	Infection due to *Aspergillus*	Chest X-ray Bronchoscopy BAL BAL Cultures	Death
Nasim et al. [8]	35/M	Infection due to *Aspergillus Fumigatus*	Chest X-ray Chest CT Bronchoscopy BAL CT-Guided Biopsy Surgical Resection of the Mass with Left Upper Lobectomy Culture of Surgical Biopsy Specimen	Recovery
Gupta A et al. [9]	45/M	Infection due to *Candida* *Tropicalis*	Chest X-ray Chest CT Bronchoscopy BAL BAL cultures	Death
Gupta KL et al. [11]	25/M	Pulmonary Mucormycosis	Chest X-ray Bronchoscopy Bronchial Biopsy Histological Examination of Bronchial Biopsy Specimen	Death
Navarro Vergara et al. [12]	56/F	Infection due to *Rhizomucor pusillus*	Chest X-ray Chest CT CT-guided Needle Aspiration Biopsy Culture and PCR of Biopsy Specimen	Death
Martin et al.[13]	18/M	Infection due to *Rhizopus*	Chest X-ray Chest CT Bronchoscopy BAL Thoracotomy with Left Lower Lobectomy Histological Examination of Biopsy Specimen	Recovery
Meigh et al. [14]	42/F	Infection due to *Legionella Pneumonophila serogroup 12*	Chest X-ray Sputum Cultures	Recovery
Moore et al. [15]	N/A	Infection due to *Legionella Pneumonophila*	Chest X-ray Serological Testing for *Legionella * Bronchoscopy Pleural Fluid Drainage Cultures of Bronchial Washings and Pleural Fluid	N/A
Speck et al. [16]	62/M	Infection due to *Rhodococcus equi*	Chest X-ray Chest CT Bronchoscopy BAL Bacterial Culture of Blood, Sputum, BAL and Pleural Fluid.	Recovery
Scoggin et al.[18]	45/M	Infection due to *Strongyloides stercoralis*	Chest X-ray Microscopic Examination of Transtracheal Aspirate of Sputum and Stool	Death
Ferreira et al.[19]	50/M	Infection due to *Strongyloides stercoralis*	Chest X-ray Microscopic Examination of Induced Sputum Smear	Death
Kwon et al. [20]	65/M	Infection due to Paragonimus westermani	Chest CT PET Wedge Resection of the lung Serum antibodies to *P. westermani*	Recovery
Meyer et al. [21]	33/F	Nitrofurantoin-induced Pulmonary Hemorrhage	Chest X-ray Bronchoscopy BAL Lung Biopsy	Recovery
Refabert et al. [22]	14/M	Azathioprine-induced Pulmonary Hemorrhage	Chest X-ray Brochoscopy-BAL	Recovery
Vlahakis et al. [23]	45/F	Sirolimus-induced Pulmonary Hemorrhage	Chest X-ray Chest CT Bronchoscopy BAL	Recovery
Pham et al. [24]	55/F	Sirolimus-induced Pulmonary Hemorrhage	Chest X-ray Chest CT Bronchoscopy BAL Transbronchial Biopsy Histological examination of Transbronchial Biopsy Specimen	Recovery
Sachdeva et al. [25]	26/M	Alemtuzumab-induced Pulmonary Hemorrhage	Chest X-ray Chest CT Bronchoscopy BAL	Recovery
Tahir et al. [26]	18/M	Alemtuzumab-induced Pulmonary Hemorrhage	Chest CT Bronchoscopy BAL	Death
Anandh et al. [27]	46/M	Mycophenolate sodium induced Pulmonary Hemorrhage	Chest CT Bronchoscopy BAL	Recovery
Khan et al. [28]	32/M	Kaposi Sarcoma of the Lung	Chest X-ray Bronchoscopy BAL Transbronchial Biopsy	Death
Scheetz et al. [29]	43/F	Kaposi Sarcoma of the Lung	Chest CT Thoracentesis Bronchoscopy PET scan Endobronchial Ultrasound-guided Biopsy	Recovery
Schulze et al. [30]	65/M	Metastatic Pulmonary Angiosarcoma	Chest X-ray Chest CT Bronchoscopy BAL Transesophageal Echocardiogram Left and Right Thoracotomy with Lung Biopsy Histological Examination of Biopsy Specimen obtained from Thoracotomy	Death

* BAL: Bronchoalveolar Lavage, CT: Computed Tomography, F: Female, M: Male, N/A: Not Available, PCR: Polymerase Chain Reaction, PET: Positron Emission Tomography

## Causes of hemoptysis in renal transplant recipients 

### Infections

Lung infections due to specific microorganisms present with hemoptysis in renal transplant recipients. Tuberculosis (TB) has been reported in kidney transplant recipients as an infection with predominance in lungs. Lattes et al. in an analysis of the characteristics of adult kidney recipients with TB infection reported a fatal case of hemoptysis developed during the first month of TB treatment [9].

Fungal infections have been associated with hemoptysis in renal transplant recipients. *Aspergillus species* have been associated with invasive pulmonary infection in renal transplant patients presenting with hemoptysis [7-8]. Gupta A et al. reported a renal allograft recipient who presented with bilateral lung abscesses due to fungus *Candida tropicalis* and developed massive hemoptysis [6]. 

Mucormycosis is an increasingly emerging life-threatening invasive fungal infection, especially in immunocompromised hosts [10]. This infection has been reported as a cause of hemoptysis in renal transplant recipients [11-13]. 

Additionally, other unusual pathogens have been demonstrated as causative factors of hemoptysis in renal transplant recipients. *Legionella pneumophila* has been mentioned as a causative agent of lower respiratory tract infection in renal transplant recipients complicated with hemoptysis [14,15]. 

*Rhodococcus equi*, a gram-positive coccobacillus bacterium, is another unusual microorganism associated with lung mass and endobronchial malakoplakia leading to hemoptysis in renal transplant recipients [16,17]. 

Besides, parasites have been described to cause pulmonary infection with hemoptysis in renal transplant recipients, especially in endemic countries. *Strongyloides stercoralis *pulmonary infection has been described to cause dyspnea, bilateral infiltrates and hemoptysis in renal transplant recipients [18,19]

Kwon et al. presented a case of renal transplant recipient who was admitted because of hemoptysis, a pulmonary cavity and right adrenal gland mass. Infection due to *Paragonimus westermani *was confirmed from biopsy specimens of wedge resection of the lung and adrenalectomy [20]. 

### Drug-Induced Pulmonary Hemorrhage

Therapeutic agents have been associated with pulmonary hemorrhage presenting with the symptom of hemoptysis in kidney transplant patients. Nitrofurantoin has been reported to cause diffuse infiltrates and worsening hemoptysis in a renal transplant recipient [21]. The immunosuppressive agent azathioprine has also been described as cause of pulmonary hemorrhage and hemoptysis in a renal transplant patient [22]. 

Sirolimus, also called as rapamycin, which is a macrolide compound that is utilized for preventing organ transplant rejection, has been associated with alveolar hemorrhage, lung infiltrates and hemoptysis in kidney transplant recipients [23,24]. 

Alemtuzumab, a monoclonal antibody that binds to CD52 protein on the surface of mature lymphocytes, is another drug related to pulmonary hemorrhage leading to hemoptysis in renal transplant recipients. Pulmonary hemorrhage associated with alemtuzumab induction therapy has been reported in two patients with Alport syndrome who developed acute dyspnea and hemoptysis after renal transplantation [25,26].

Anandh et al. described a 46-year-old man, renal transplant recipient, who presented with cough and hemoptysis on 1st posttransplant day. Mycophenolate sodium was thought the potential cause of hemoptysis. The withdrawal of the drug resulted in patient’s improvement [27].

### Malignancies

Rare types of malignancies have been reported as underlying causes of hemoptysis in renal transplant patients. Kaposi sarcoma is a well established human herpesvirus-8 (HHV-8) driven complication of renal transplantation and immunosuppression with lung involvement. It is an uncommon malignancy thought to arise from precursor endothelial cells in a multicentric pattern resulting in tumor of mixed vascular and fibroblastic origin [28]. Kaposi sarcoma of the lung has been described to cause pulmonary hemorrhage, bilateral pulmonary infiltrates and hemoptysis [28] and acute hypoxic respiratory failure and hemoptysis [29] in renal transplant recipients. 

Pulmonary metastatic angiosarcoma is a rare cause of hemorrhage, traditionally showing multiple nodules on chest CT. Pulmonary metastatic angiosarcoma leading to hemoptysis has been diagnosed in two cases of renal transplant recipients [30].

## Investigations for hemoptysis

### Imaging

Chest X-ray is the initial diagnostic modality for the investigation of underlying cause of hemoptysis in renal transplant recipients providing useful information about the location and characteristics of the lung lesions [6-8, 9, 11-13, 15-17, 19-122, 24-27, 29]. In several cases, chest X-ray guides the physicians to possible diagnoses without the need of chest CT [5, 8, 11-13, 19, 22]. Chest CT is a more sensitive imaging tool for the detection of pulmonary abnormalities responsible for hemoptysis in renal transplant patients, facilitating the identification of the area for bronchoscopic approach and narrowing the differential diagnosis of hemoptysis [8-9, 12-13, 16, 18, 21, 24-29]. In addition, chest CT allows the image-guided needle aspiration biopsy of thoracic lesions, establishing the final cause of hemoptysis [8, 12].

Positron Emission Tomography (PET) has been used to differentiate benign lung lesions from nonbenign abnormalities in cases that the suspicion of malignancy is high [21,31]. Transesophageal echocardiogram is another imaging tool that has been utilized in a case of an angiosarcoma of the right atrium, metastatic to bones and lungs, causing hemoptysis in a kidney transplant recipient [30].

### Bronchoscopy

The role of traditional methods to diagnose lung infections is limited in immunocompromised renal transplant recipients [31]. Suspected lung infection is the most common indication for bronchoscopy among solid organ recipients. Bronchoscopy has been used in the diagnostic approach of hemoptysis in renal transplant recipients in order to provide inspection of bronchial tree for discovering potential site of active bleeding and to obtain bronchoalveolar lavage (BAL), bronchial washings, bronchial brushings, bronchial biopsies, endobronchial ultrasound-guided fine needle aspiration (EBUS-FNA) biopsies of mediastinal lesions and transbronchial biopsy (TBB) for histological and microbiological examination [7-13, 11, 13, 15-16, 18, 22, 24-30].

### Thoracentesis and Pleural Drainage

Thoracentesis and drainage of pleural fluid have been performed in some cases of hemoptysis, that pulmonary lesions are accompanied by pleural effusion, in order to examine the fluid for pathogenic microorganisms or abnormal cytology [15,30].

### Microbiological Evaluation-Serological Testing

Microbiological evaluation of different specimens and body fluids, with microscopic examination, culture and evaluation with polymerase chain reaction (PCR), has an important role in the investigation of hemoptysis in renal transplant recipients, confirming or excluding specific infections [6-9, 12, 14-16, 18-20, 22, 24-26, 28-29]. Serological testing for antibodies (IgG and IgM) to specific microorganisms has also been used for the diagnosis of underlining lung infections in renal transplant recipients with hemoptysis [15, 21].

### Surgical Procedures

Surgical procedures have been utilized for the diagnostic approach of hemoptysis in renal transplant recipients in cases when investigation with imaging, bronchoscopic techniques and microbiology/ serology testing did not reveal a diagnosis or in order to confirm a controversial diagnosis. Thoracotomy with lobectomy, thoracotomy with lung biopsy and video assisted thoracoscopic wedge resection of the lung have been used for obtaining lung tissue and establish the underlying diagnosis of hemoptysis [8, 13, 20-21]. 

### Discussion

Ηemoptysis in renal transplant recipients, as immunocompromised patients, can occur in the context of infections (tuberculosis, mycetoma, invasive pulmonary aspergillosis, necrotizing parenchymal pneumonia, parasitic infection) [5-20, 32]. Hemoptysis can also occur in the context of post-transplant malignancy due to immunosuppression (bronchial cancer, pulmonary metastases from other primary site) [28-30, 33], in the context of potential drug toxicity [21-26, 34], use of anticoagulants or antiplatelet agents [34], pulmonary embolism [35] or bronchiectasis due to recurrent pulmonary infections [36]. According to the existing literature, it cannot be concluded whether one or another reasons could lead to better or worse outcomes. Table 2 shows potential conditions leading to hemoptysis in renal transplant recipients.

**Table 2.** Potential causes of hemoptysis in renal transplant recipients.

**Pulmonary Infections**	Tuberculosis, Mycetoma, Invasive Pulmonary Aspergillosis, Bacterial Pneumonia, Necrotizing Parenchymal Pneumonia, Parasitic infection
**Posttransplant malignancies**	Bronchial cancer, Kaposi Sarcoma, Angiosarcoma and Pulmonary Metastases from other primary site
**Drug toxicity**	Nitrofurantoin, Alemtuzumab, Azathioprine, Sirolimus, Mycophenolate sodium
**Coagulation Disorders**	Anticoagulants or antiplatelet agents
**Other Pulmonary Causes**	Pulmonary Embolism, Bronchiectasis

Physicians should keep in mind that the course of hemoptysis is unpredictable. Thus, the management is standardized, including several steps. The first steps include confirming the diagnosis of hemoptysis and assessment of its severity. The next step should focus to localize the site and find the cause of hemoptysis. Finally, the most appropriate therapeutic approach should be decided. It is of great importance, in case of severe hemoptysis, locating the site of bleeding and administering specific treatment including maintenance of free airways, to be performed simultaneously with identifying the cause of bleeding. Therefore, chest-X-ray, chest CT scan and bronchoscopy are essential procedures in the diagnostic algorithm. Coagulation tests are also useful in case of suspection of coagulation disorder [37]. 

This review has some limitations. Our results are limited by the quality and extent of the data in the reports. More specifically, case reports are unchecked, and while they can recommend hypotheses, they are not able to confirm robust conclusions. However, clinicians should be aware of even the few number of cases reported in the literature suggesting rare cases of hemoptysis in renal transplant recipients. 

## Conclusions

In conclusion**, **hemoptysis is an important respiratory symptom in renal transplant patients. Clinical entities related to hemoptysis in renal transplant patients are different from these in common population. Opportunistic lung infections, drug-induced pulmonary hemorrhage and rare types of malignancy should be taken into account in the differential diagnosis of hemoptysis in these patients. Imaging with chest X-ray and CT, bronchoscopic techniques and microbiological evaluation of body fluids are important diagnostic modalities. Surgical procedures are used less frequently for diagnosis and management, especially when hemoptysis persists or becomes severe despite conservative management.

## References

[ref1] Lee BR, Yu JY, Ban HJ, et al. Analysis of patients with hemoptysis in a tertiary referral hospital. Tuberc Respir Dis (Seoul). 2012;73(2):107-14. doi: 10.4046/trd.2012.73.2.107. 23166543 PMC3492374

[ref2] Drent M, Wessels S, Jacobs JA, Thijssen H. Association of diffuse alveolar haemorrhage with acquired vitamin K deficiency. Respiration. 2000;67(6):697. doi: 10.1159/000056305. 11124658

[ref3] Ikeda M, Tanaka H, Sadamatsu K. Diffuse alveolar hemorrhage as a complication of dual antiplatelet therapy for acute coronary syndrome. Cardiovasc Revasc Med 2011;12:407. 21514251 10.1016/j.carrev.2011.03.003

[ref4] Chen BC, Sheth NR, Dadzie KA, et al. Hemodialysis for the treatment of pulmonary hemorrhage from dabigatran overdose. Am J Kidney Dis 2013; 62:591. 23597859 10.1053/j.ajkd.2013.02.361

[ref5] Schlageter M, Jahn KD, Tzankov A, et al. An unexpected cause of diffuse alveolar hemorrhage in a kidney transplant patient. Respiration. 2014;87(6):504-7. doi: 10.1159/000358566. 24732422

[ref6] Lattes R, Radisic M, Rial M, Argento J, Casadei D. Tuberculosis in renal transplant recipients. Transpl Infect Dis. 1999;1(2):98-104. doi: 10.1034/j.1399-3062.1999.010203.x. 11428977

[ref7] Ardalan MR, Ansarin K, Hejazi E, Nazemieh M, Safa J. Aspergillosis after Renal Transplantation. Saudi J Kidney Dis Transplant 2005;16(3):330-333. 17642801

[ref8] Nasim A, Baqi S, Zeeshan SM, Aziz T. Chronic necrotizing pulmonary aspergillosis in a renal transplant recipient. J Pak Med Assoc. 2011;61(12):1242-4. 22355978

[ref9] Gupta A, Bhowmik DM, Dogra PM, Mendonca S, Gupta A. Candida lung abscesses in a renal transplant recipient. Saudi J Kidney Dis Transpl. 2013;24(2):315-7. doi: 10.4103/1319-2442.109589. 23538356

[ref10] Song Y, Qiao J, Giovanni G, et al. Mucormycosis in renal transplant recipients: review of 174 reported cases. BMC Infect Dis. 2017;17(1):283. doi: 10.1186/s12879-017-2381-1. 28420334 PMC5395857

[ref11] Gupta KL, Khullar DK, Behera D, Radotra BD, Sakhuja V. Pulmonary mucormycosis presenting as fatal massive haemoptysis in a renal transplant recipient. Nephrol Dial Transplant. 1998;13(12):3258-60. doi: 10.1093/ndt/13.12.3258. 9870509

[ref12] Navarro Vergara DI, Barragán Pola G, Bonifaz A, Núñez Pérez-Redondo C, Choreño García O, Cicero Sabido R. Mucormicosis pulmonar en un paciente con trasplante renal y hemoptisis incoercible [Pulmonary mucormycosis in a patient with kidney transplant and uncontrolled haemoptysis]. Rev Iberoam Micol. 2017;34(4):233-236. doi: 10.1016/j.riam.2017.03.005. 28757006

[ref13] Martin MS, Smith AA, Lobo M, Paramesh AS. Successful Treatment of Recurrent Pulmonary Mucormycosis in a Renal Transplant Patient: A Case Report and Literature Review. Case Rep Transplant. 2017;2017:1925070. doi: 10.1155/2017/1925070. PMC5366780 28386509

[ref14] Meigh RE, Makin T, Scott MH, Hart CA. Legionella pneumophila serogroup 12 pneumonia in a renal transplant recipient: case report and environmental observations. J Hosp Infect. 1989;13(3):315-9. doi: 10.1016/0195-6701(89)90013-3. 2567762

[ref15] Moore EH, Webb WR, Gamsu G, Golden JA. Legionnaires’ disease in the renal transplant patient: clinical presentation and radiographic progression. Radiology. 1984;153(3):589-93. doi: 10.1148/radiology.153.3.6387784. 6387784

[ref16] Speck D, Koneth I, Diethelm M, Binet I. A pulmonary mass caused by Rhodococcus equi infection in a renal transplant recipient. Nat Clin Pract Nephrol. 2008;4(7):398-403. doi: 10.1038/ncpneph0833. 18506169

[ref17] Lococo F, Montanari G, Mengoli MC, Ferrari F, Spagnolo P, Rossi G. Hemoptysis and Progressive Dyspnea in a 67-Year-Old Woman with History of Renal Transplantation. Am J Respir Crit Care Med. 2016;193(6):e12-3. doi: 10.1164/rccm.201511-2134IM. 26681504

[ref18] Scoggin CH, Call NB. Acute respiratory failure due to disseminated strongyloidiasis in a renal transplant recipient. Ann Intern Med. 1977;87(4):456-8. doi: 10.7326/0003-4819-87-4-456. 334007

[ref19] Ferreira CJ, Silva DA, Almeida PH, et al. Fatal disseminated strongyloidiasis after kidney transplantation. Rev Soc Bras Med Trop. 2012;45(5):652-4. doi: 10.1590/s0037-86822012000500022. 23152354

[ref20] Kwon YS, Lee HW, Kim HJ. Paragonimus westermani infection manifesting as a pulmonary cavity and adrenal gland mass: A case report. J Infect Chemother. 2019;25(3):200-203. doi: https://doi.org/S1341-321X(18)30255-1. 30213500 10.1016/j.jiac.2018.08.005

[ref21] Meyer MM, Meyer RJ. Nitrofurantoin-induced pulmonary hemorrhage in a renal transplant recipient receiving immunosuppressive therapy: case report and review of the literature. J Urol. 1994;152(3):938-40. doi: 10.1016/s0022-5347(17)32615-0. 8051759

[ref22] Refabert L, Sinnassamy P, Leroy B, Fauroux B, de Blic J, Bensman A. Azathioprine-induced pulmonary haemorrhage in a child after renal transplantation. Pediatr Nephrol. 1995;9(4):470-3. doi: https://doi.org/10.1007/ BF00866729. 7577411 10.1007/BF00866729

[ref23] Vlahakis NE, Rickman OB, Morgenthaler T. Sirolimus-associated diffuse alveolar hemorrhage. Mayo Clin Proc. 2004;79(4):541-5. doi: 10.4065/79.4.541. 15065619

[ref24] Pham PT, Pham PC, Danovitch GM, Ross DJ, Gritsch HA, Kendrick EA, Singer J, Shah T, Wilkinson AH. Sirolimus-associated pulmonary toxicity. Transplantation. 2004;77(8):1215-20. doi: 10.1097/01.tp.0000118413.92211.b6. 15114088

[ref25] Sachdeva A, Matuschak GM. Diffuse alveolar hemorrhage following alemtuzumab. Chest. 2008;133(6):1476-1478. doi: 10.1378/chest.07-2354. 18574290

[ref26] Tahir W, Hakeem A, Baker R, Ahmad N. Diffuse Alveolar haemorrhage: a fatal complication after alemtuzumab induction therapy in renal transplantation. Transplant Proc. 2015;47(1):151-4. doi: 10.1016/j.transproceed.2014.10.037. 25645795

[ref27] Anandh U, Marda S. Unusual Case of Acute Lung Injury in a Renal Allograft Recipient. Indian J Nephrol. 2017;27(3):215-217. doi: 10.4103/0971-4065.202827. 28553043 PMC5434689

[ref28] Khan GA, Klapper P. Pulmonary haemorrhage following renal transplantation. Thorax. 1995;50(1):98-99. doi: 10.1136/thx.50.1.98. 7886663 PMC473733

[ref29] Scheetz S, Pandey D, Pesavento TE, Singh P. Pulmonary Presentation of Kaposi-Sarcoma in a Renal Transplant Recipient. Cureus. 2020;12(1):e6719. doi: 10.7759/cureus.6719. 32104640 PMC7032608

[ref30] Schulze R, Schulze M, Salomon-Dani A, et al. Rezidivierende Hämoptysen. 65-jähriger nierentransplantierter Patient mit beidseitigen Thoraxwandtumoren [Recurrent hemoptysis. 65-year-old post-kidney transplant patient with bilateral thoracic wall tumors]. Internist (Berl). 2001;42(1):119-23. doi: 10.1007/s001080050729. 11271612

[ref31] Eriksson BM, Dahl H, Wang FZ, et al. Diagnosis of pulmonary infections in immunocompromised patients by fiber-optic bronchoscopy with bronchoalveolar lavage and serology. Scand J Infect Dis. 1996;28(5):479-85. doi: 10.3109/00365549609037944. 8953678

[ref32] Wilson WR, Cockerill FR 3rd, Rosenow EC 3rd. Pulmonary disease in the immunocompromised host (2). Mayo Clin Proc. 1985;60(9):610-31. doi: 10.1016/s0025-6196(12)60985-9. 3894817

[ref33] Rousseau-Gazaniol C, Fraboulet S, Couderc LJ, et al. Lung cancer in renal transplant recipients: A case-control study. Lung Cancer. 2017 Sep;111:96-100. doi: 10.1016/j.lungcan.2017.07.011. 28838407

[ref34] Serifoglu I, Er Dedekarginoglu B, Ayvazoglu Soy EH, et al. Causes of Hemoptysis in Renal Transplant Patients. Exp Clin Transplant. 2018;16 Suppl 1(Suppl 1):70-74. doi: 10.6002/ect.TOND-TDTD2017.O30. 29527996

[ref35] Küpeli E, Ulubay G, Doğrul I, Birben Ö, Seyfettin P, Özsancak Uğurlu A, Öner Eyüboğlu F, Haberal M. Long-term risk of pulmonary embolism in solid-organ transplant recipients. Exp Clin Transplant. 2015 Apr;13 Suppl 1:223-7. 25894159

[ref36] Dury S, Colosio C, Etienne I, et al. Bronchiectasis diagnosed after renal transplantation: a retrospective multi-center study. BMC Pulm Med. 2015;15:141.doi: 10.1186/s12890-015-0133-9. 26545860 PMC4636796

[ref37] Tsoumakidou M, Chrysofakis G, Tsiligianni I, Maltezakis G, Siafakas NM, Tzanakis N. A prospective analysis of 184 hemoptysis cases: diagnostic impact of chest X-ray, computed tomography, bronchoscopy. Respiration. 2006;73(6):808-14. doi: 10.1159/000091189 16446530

